# Effects of Treatment Length and Chat-Based Counseling in a Web-Based Intervention for Cannabis Users: Randomized Factorial Trial

**DOI:** 10.2196/jmir.9579

**Published:** 2018-05-08

**Authors:** Benjamin Jonas, Marc-Dennan Tensil, Peter Tossmann, Evelin Strüber

**Affiliations:** ^1^ Delphi Gesellschaft für Forschung, Beratung und Projektentwicklung Berlin Germany; ^2^ Federal Centre for Health Education Cologne Germany

**Keywords:** cannabis, Internet, counseling, random allocation

## Abstract

**Background:**

Digital interventions show promise in reducing problematic cannabis use. However, little is known about the effect of moderators in such interventions. The therapist-guided internet intervention Quit the Shit provides 50 days of chat-based (synchronous) and time-lagged (asynchronous) counseling.

**Objective:**

In the study, we examined whether the effectiveness of Quit the Shit is reduced by shortening the program or by removing the chat-based counseling option.

**Methods:**

We conducted a purely Web-based randomized experimental trial using a two-factorial design (factor 1: real-time-counseling via text-chat: yes vs no; factor 2: intervention duration: 50 days vs 28 days). Participants were recruited on the Quit the Shit website. Follow-ups were conducted 3, 6, and 12 months after randomization. Primary outcome was cannabis-use days during the past 30 days using a Timeline Followback procedure. Secondary outcomes were cannabis quantity, cannabis-use events, cannabis dependency (Severity of Dependence Scale), treatment satisfaction (Client Satisfaction Questionnaire), and working alliance (Working Alliance Inventory-short revised).

**Results:**

In total, 534 participants were included in the trial. Follow-up rates were 47.2% (252/534) after 3 months, 38.2% (204/534) after 6 months, and 25.3% (135/534) after 12 months. Provision of real-time counseling (factor 1) was not significantly associated with any cannabis-related outcome but with higher treatment satisfaction (*P*=.001, *d*=0.34) and stronger working alliance (*P*=.008, *d*=0.22). In factor 2, no significant differences were found in any outcome. The reduction of cannabis use among all study participants was strong (*P*<.001, *d*≥1.13).

**Conclusions:**

The reduction of program length and the waiver of synchronous communication have no meaningful impact on the effectiveness of Quit the Shit. It therefore seems tenable to abbreviate the program and to offer a self-guided start into Quit the Shit. Due to its positive impact on treatment satisfaction and working alliance, chat-based counseling nevertheless should be provided in Quit the Shit.

**Trial Registration:**

International Standard Randomized Controlled Trial Number ISRCTN99818059; http://www.isrctn.com/ISRCTN99818059 (Archived by WebCite at http://www.webcitation.org/6uVDeJjfD)

## Introduction

Cannabis is the most widely used illegal drug in Europe with a last-year prevalence of 14% among young adults and around 1% daily or almost daily users [1]. In 2015, cannabis use of 550,000 adults in Germany was clinically relevant [2].

In the past years, several online interventions targeting individuals with cannabis use disorder (CUD) were made available to the public in Europe [3]. The guided program “Quit the Shit” (QTS) is the only intervention that has been tested in a randomized controlled trial (RCT) [4-7]. Besides the Australian intervention “Reduce your use,” QTS is currently the only evidence-based internet intervention targeting cannabis users that is freely available for the public [8].

As part of the drug prevention website drugcom, QTS is operated by the German Federal Centre for Health Education (BZgA) since 2004 and is one of the components of the prevention strategy for CUD of the BZgA [4,9]. With 50 days of individual counseling by trained therapists, QTS offers more intense support than most other evaluated interventions related to cannabis use [5,6]. Free and anonymous QTS provides direct (synchronous) and time-lagged (asynchronous) counseling. Synchronous counseling via live chat is mainly offered during admission and termination of the intervention. Asynchronous support is delivered by weekly feedbacks on participants’ input in the cannabis use diary and exercises of QTS. The intervention is described in detail in the Methods section.

To account for the increasing demand of QTS and to make the program less dependent on prescheduled chat counseling, our interest was to test whether shortening the intervention and whether eliminating chat-based counseling has negative impact on the effectiveness of QTS. A shorter version of QTS would presumably reduce the counselor’s effort per client and thereby allow increasing the number of participants. Moreover, a program without synchronous communication would be more flexible because participants would not be dependent on prescheduled chats to start the intervention. However, those changes should not significantly reduce the effectiveness of QTS or decrease the user satisfaction or working relation between the counselor and client.

Although internet interventions targeting substance-related disorders have shown to be effective, little is known about moderators of their effectiveness [6,10]. Despite some indication in favor of longer alcohol-related treatments, evidence is still mixed [10]. For internet interventions targeting CUD, there is no such evidence in either direction [6]. Although internet interventions based on synchronous interaction have shown to be generally effective [11], their effects compared with asynchronous-only counseling were not investigated systematically, pointing to a need of further research in this area [12]. Evidence from an earlier meta-analysis indicated no better performance of either synchronous or asynchronous mode of interaction [13].

To secure the planned changes to QTS with empirical evidence, we therefore examined whether shortening the intervention or eliminating chat-based counseling has negative impact on the program. The main outcome was cannabis use frequency during the past 30 days. Secondary outcomes were cannabis quantity, cannabis use events, cannabis dependency, treatment satisfaction, and working alliance.

## Methods

### Study Design

We conducted a purely Web-based pragmatic randomized experimental trial using a two-factorial design. In the study, participants were allocated to 1 of the 4 versions of QTS (Table 1). In the first experimental factor, we compared intervention versions containing chat-based counseling with versions which only consisted of counseling via asynchronous communication channels. In the second factor, program versions with the regular length of 50 days were compared with versions with the reduced duration of 28 days.

The study was conducted on the website of the intervention. Trial participants were directly recruited from all individuals who were interested in signing up for QTS. At the beginning of the program registration, potential participants were informed about the study. A PDF file containing all relevant study details was offered for download and in the confirmation email for study participants. The PDF file is included in the Multimedia Appendix 1. Individuals who were willing to participate were asked to register and provide their informed consent by checking an “I agree to participate” checkbox. The study outcomes were included in the regular registration questionnaire of QTS. Users of the intervention who opted not to participate in the study or who did not meet the eligibility criteria had full access to the regular version of the intervention and were not included in any follow-up surveys.

After registration, study participants were to choose an appointment from a schedule provided by QTS. By logging into the program at this appointment, participants were randomized automatically to 1 of the 4 intervention versions and directly forwarded into the program. Neither the researchers nor the counselors could influence or predict the randomization result. Participants were blind to the results of the randomization because they only received detailed information about the program version they were allocated to. None of the intervention versions underwent any changes during the evaluation process.

Follow-up surveys were conducted in online questionnaires 3, 6, and 12 months after randomization. Each follow up participation was compensated for with a gift voucher for a major internet-based retailer worth 10 euros.

The study was approved by the ethics committee of the Department of Applied Human Sciences at the University of Magdeburg-Stendal (Ref 4973-35) and was registered with ISRCTN (ISRCTN99818059). Note: In the registry, the study design was erroneously declared as RCT. The right indication, however, should have been randomized factorial trial.

**Table 1:** Experimental design.

Experimental design		Factor 2: Intervention length	
		50 days	28 days
**Factor 1: Chat-based counseling**			
	Yes	Version 1	Version 2
	No	Version 3	Version 4

### Measures

Cannabis use was measured using the Timeline Followback method (TLFB) [14]. In the TLFB form, participants had to indicate the number of joints, bongs, and other cannabis use for each day in the past 30 days in a calendar. They were encouraged to use anchor events such as birthdays, appointments, or holidays to get a better orientation. The number of cannabis use days and the number of use events (ie, the sum of joints, bongs, and other cannabis use) were derived from the input from the TLFB form. To measure the cannabis quantity in grams, participants were asked to estimate this sum over the past 30 days.

Cannabis dependence was measured by the German version of the Severity of Dependence Scale (SDS) [15,16], changing the reference period from 12 to 3 months. Participants with a SDS score of at least 4 points were categorized as cannabis dependent [17]. Effects on treatment satisfaction were measured using the German version of the Client Satisfaction Questionnaire (CSQ-8) [18,19]. Data on the working alliance between counselor and participant were collected using the German version of the Working Alliance Inventory-short revised (WAI-sr) [20,21]. The CSQ-8 and WAI-sr were not listed in the study registry, as they were introduced after registering the study.

As a measure of the program usage, we tracked the number of logins and the duration of chat-based counseling for each participant.

### Study Criteria

To be eligible for the study, individuals had to be at least 18 years old and to be first-time users of the intervention. Exclusion criteria were alcohol use disorder operationalized by a score of at least 3 in the CAGE [22], diagnosed psychotic or bipolar disorder based on self-report, current use of other illicit drugs on more than 4 days during the last 30 days, and suicidal thoughts as measured by selected items of Becks Scale for Suicide Ideation [23]. Individuals who displayed suicidal thoughts were given detailed information on suitable psychosocial support options such as telephone helplines or local institutions.

### Interventions

#### Version 1 (Regular Quit the Shit)

The first intervention version of the trial was identical to the regular QTS program and therefore was 50 days long. QTS in general is based on the principles of self-regulation and self-control [24]. The weekly feedbacks are based on the solution-focused approach [25] and motivational interviewing [26]. Therefore, the participants’ responsibility to achieve personal change is accentuated, current personal developments and achievements are reviewed, and clear recommendations for further steps concerning the solution of problems are given. After the registration, the intervention comprises 3 consecutive phases:

First, admission takes place during a prescheduled appointment in a one-to-one chat with a counselor of QTS. The chat takes 50 min and is mandatory to enter QTS. It aims at clarifying the situation of the client and helps determine individual consumption goals and coping strategies. Usually, each user stays with the same counselor throughout the whole program.

Second, after the admission chat, the login area of QTS is activated. It contains a diary where participants are required to write down all relevant aspects of their cannabis use over the whole duration of 50 days. Moreover, the login area includes coping exercises, for example, aiming to develop control strategies, enhancing quality of life, or balancing the pros and cons of using cannabis. Once a week, participants receive detailed feedback by their counselor on their entries in diary and exercises. Depending on the involvement of each participant, up to 7 feedbacks are given. They discuss the current cannabis use, the psychosocial situation, and the counseling process as such.

Third, at the end of the program, clients are invited to a concluding chat, where the initial consumption goals and the applied control strategies are reflected. If necessary, participants are referred to local addiction counseling or therapy.

#### Version 2

Version 2 is an abbreviated variant of the original intervention and thus is only usable for 28 days. As the counselor feedback is timed weekly, version 2 therefore includes only 4 feedbacks as opposed to 7 in version 1. Apart from that, there are no differences.

#### Version 3

In contrast to versions 1 and 2, this variant of QTS does not offer synchronous communication between the counselor and client. Instead of starting the intervention via live chat, users of version 3 are to describe their current situation and their program goals in a self-guided tour. In open text boxes, participants are requested to outline their cannabis use, their personal pros and cons for using cannabis, and their ideas of what or who might help them to reduce their use. After concluding the self-guided tour, the login area, as described above, is activated automatically. The first counselor feedback 1 week later refers to the first-week entries in diary and to the input in the self-guided tour.

Like version 1, this variant of QTS is 50 days long and therefore provides up to 7 weekly feedbacks. Instead of a live chat at the end of the program, the last feedback is used to summarize the progress made during participation, to reconsider the working strategies, and to determine whether further professional help is required.

#### Version 4

**Figure 1:**
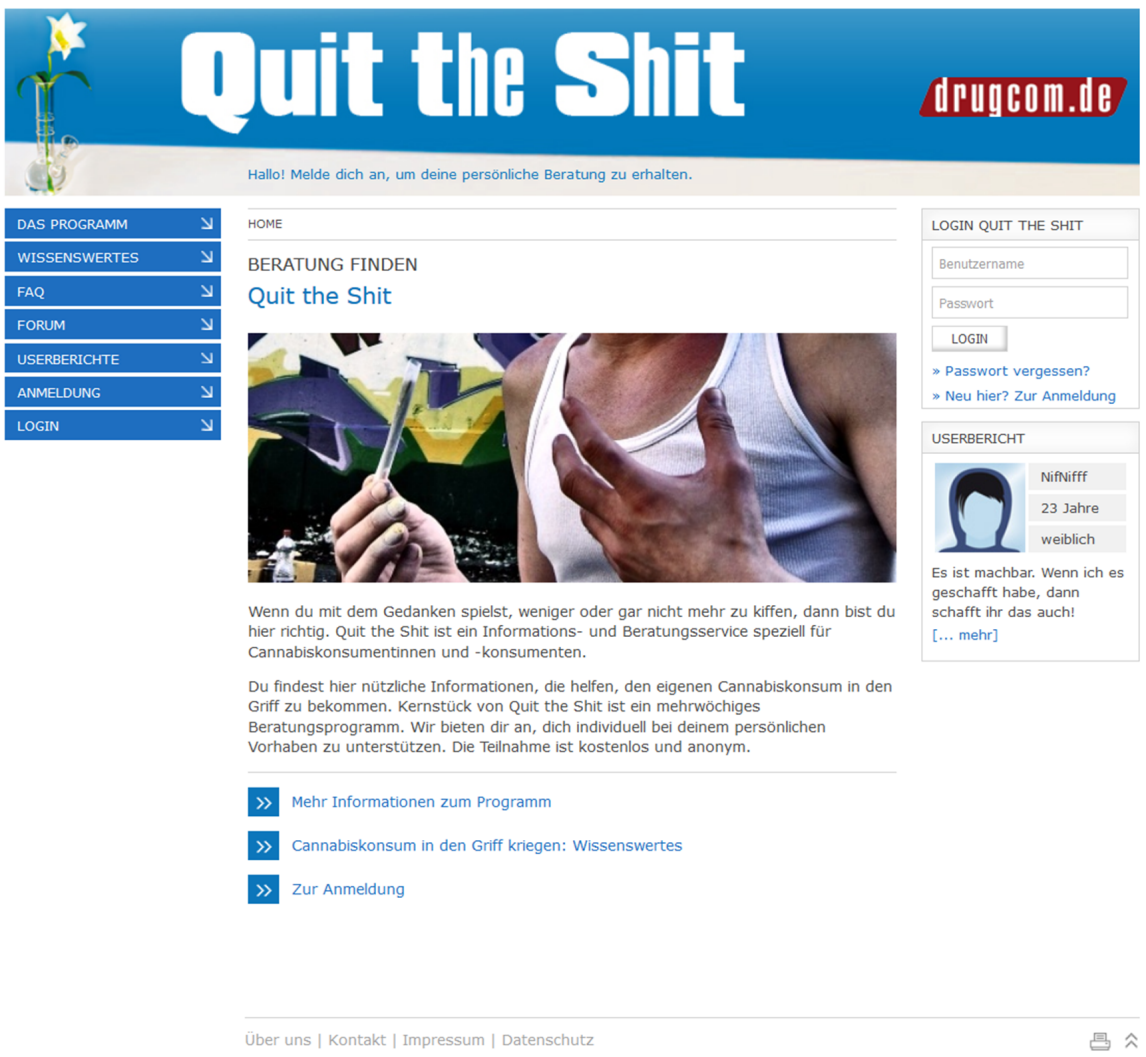
Home page of “Quit the Shit” (QTS).

Identical to version 3, version 4 does not include chat-based interaction and instead consists of the same self-guided admission procedure as described above. The only difference in comparison with version 3 is the shortened duration of 28 days. For a comparison of the intervention versions, see Table 2. A screenshot of the QTS home page is shown in Figure 1. Furthermore, a screenshot of the program diary is provided in Multimedia Appendix 2.

### Statistical Analysis

Generalized estimating equations were used to examine the effects of the experimental factors on all cannabis-related study outcomes (ie, cannabis use and cannabis dependence). Treatment-related satisfaction and working alliance only were collected at the first follow-up. To measure the effects on these outcomes, we therefore used generalized linear models. In a first step of data analysis, we included both factors (Table 1) as main effects, the interaction of each factor with time, the interaction of both factors with each other, and the 3-way interaction of both factors and time in each model. Moreover, we tested whether group differences at baseline and group differences in follow-up participation moderated the factorial effects on each study outcome. If statistically significant, the respective term and its interaction with each factor were included in the models. Otherwise, it was not considered in the effectiveness testing. We assumed factorial effects on each outcome if the interaction between the respective factor and the time variable was statistically significant. To measure the overall development for each outcome, the main effect of time was examined.

The study was powered to detect a difference between factor levels in the reduction of cannabis use frequency (ie, consumption days in the past 30 days) of at least 20%. We utilized means and SDs of an earlier trial [4] to compute the associated effect size [27]. In that trial, participants of the regular QTS intervention reduced their cannabis use by approximately 14 days (SD=12.0). We, therefore, aimed to detect a difference between 14 days and 11.2 days [14 days × (1−0.2)] reduction. To detect the corresponding effect size of f=0.12, a total sample of n=552 is required (n=138 for each cell of the factorial design; two-sided alpha=.05; power=0.80).

We conducted intention-to-treat analyses, including all randomized participants according to their group allocation. Missing data were estimated by multiple imputations. We performed 50 imputations. The effectiveness results of the imputed datasets were compared with the results of the nonimputed dataset (completer-only analyses).

Logistic regression analyses were conducted to compare study participants with study nonparticipants (ie, regular users of QTS) at baseline, to compare factor levels at baseline, and to determine whether baseline measures were predicting follow-up participation.

All analyses were conducted with R 3.4.1 (R Foundation for Statistical Computing, Vienna, Austria) [28], utilizing the following commands: glm [28], geeglm [29], and des [30]. Multiple imputations were estimated with R’s mice package [31].

**Table 2:** Comparison of the interventions.

Characteristics	Version 1^a^	Version 2	Version 3	Version 4
Duration	50 days	28 days	50 days	28 days
Chat-based counseling	Yes	Yes	No	No
Registration (baseline)	Registration questionnaire	Registration questionnaire	Registration questionnaire	Registration questionnaire
Admission to intervention	Live chat (~50 min)	Live chat (~50 min)	Self-guided	Self-guided
Cannabis use diary and exercises	Up to 50 days	Up to 28 days	Up to 50 days	Up to 28 days
Weekly feedback	Up to 7	Up to 4	Up to 7	Up to 4
Conclusion of intervention	Live chat (~30 min)	Live chat (~30 min)	The last weekly feedback	The last weekly feedback

^a^Version 1 is identical to the original Quit the Shit intervention.

## Results

### Flow of Participants

**Figure 2:**
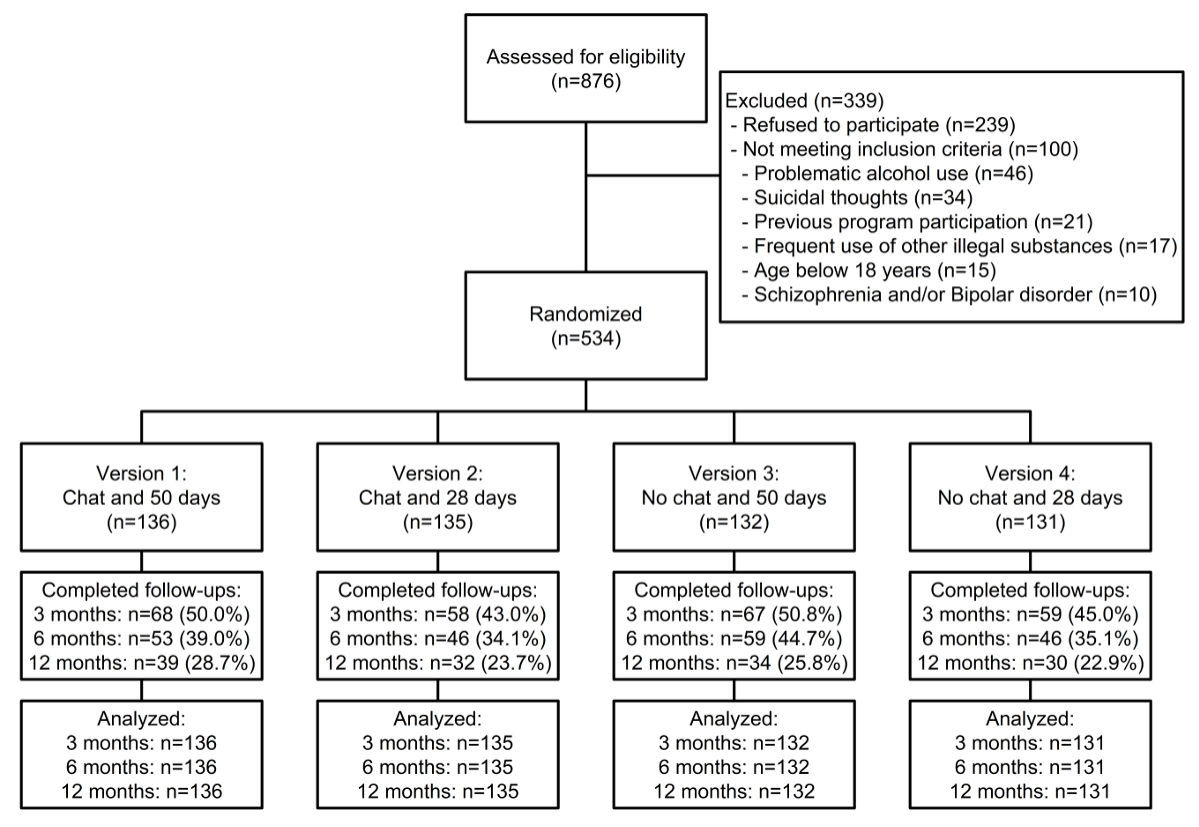
CONSORT (Consolidated Standards of Reporting Trials) flow diagram of participants.

During the study, 876 individuals accessed the baseline questionnaire of QTS and therefore were assessed for eligibility (Figure 2). In total, 339 individuals did not take part in the study, mainly because they refused to participate (n=239). In total, 100 persons did not meet all study criteria, primarily due to problematic alcohol use (n=46), suicidal thoughts (n=34), or because they had used QTS before (n=21). The randomization of the 534 participants resulted in similar-sized study groups. In total, 252 individuals provided data at the first follow-up, 204 at the second, and 135 participants filled out the last follow-up survey 12 months after randomization, resulting in follow-up rates of 47.2%, 38.2%, and 25.3%, respectively.

Follow-up participation was significantly predicted by a higher age (OR 1.03, 95% CI 1.00-1.06, *P*=.02, *d*=0.19), lower cannabis quantity (OR 0.99, 95%-CI: 0.98-1.00, *P*=.004, *d*=0.25), higher school education (OR 1.76, 95% CI 1.35-2.30, *P*<.001), and higher number of logins during program participation (OR 1.05, 95% CI 1.04-1.06, *P*<.001, *d*=0.88). The allocation to either factor level, however, was no significant predictor for follow-up participation (Factor 1: OR 0.43, 95% CI 0.18-0.99, *P*=.05; Factor 2: OR 1.05, 95% CI 0.71-1.54, *P*=.82).

### Sample Description

Baseline characteristics and program usage of the study participants are shown in Table 3. The majority of participants were male (65.7%) and had a high educational level with 64.7% attending or having successfully finished the highest German secondary school type (“Gymnasium”) [32]. As expected, cannabis use was high with only few abstinent days during the last month.

Individuals who used the regular QTS intervention without taking part in the study (n=339, see Figure 2) had comparable values in most baseline variables. The only exception was found in the cannabis use days, which was slightly higher among individuals who were excluded from the study (OR 1.02, 95% CI 1.01-1.04, *P*=.01, *d*=0.19).

Except for a small age-related difference in factor 2 (OR 0.97, 95% CI 0.95-0.996, *P*=.03, *d*=0.20), randomization resulted in similar groups. As expected, the duration of chat-based counseling differs within factor 1 (OR 1.08, 95% CI 1.07-1.09, *P*<.001, *d*=2.72) with 105.3 min of counseling chats among participants who were in the chat-based versions of QTS. In factor 2, individuals who used the longer versions logged in significantly more often than participants allocated to the shorter versions (OR 1.01, 95% CI 1.01-1.02, *P*=.001, *d*=0.32). However, with 31.6 as opposed to 23.2 logins, that number is disproportionally low regarding the difference of 50 versus 28 days program length.

### Comparison of Effectiveness

The effectiveness results reveal no significant differences between factor levels in any of the cannabis-related outcomes both in the imputed and in the nonimputed dataset (Tables 4 and 5; Multimedia Appendix 3). In working alliance and treatment satisfaction, however, significant differences with small effect sizes were found favoring chat-based communication.

None of the 3-way interactions (factor 1×factor 2×time) on the cannabis-related outcomes were significant, suggesting no relevant effectiveness differences between particular program versions (eg, versions 1 and 4). In the imputed dataset, this also applies to the two-way interactions (factor 1×factor 2) on working alliance and treatment satisfaction, which were only measured during the first follow-up (see Table 5). However, in the nonimputed dataset, we found significant two-way interactions on working alliance and treatment satisfaction (WAI: beta=−.55, CI −1.00 to −0.10, *P*=.02; CSQ: beta=−.41, CI −0.72 to −0.10, *P*=.01; Multimedia Appendix 3). The highest WAI and CSQ ratings were identified in the short version including chat-based counseling and the lowest ratings were found in the short version without chat-based counseling (see Multimedia Appendix 3).

Significant and strong time effects indicate a great overall reduction of cannabis use and use-related symptoms. The strongest reduction in the imputed dataset is found in the cannabis use days (beta=−.34, CI −0.45 to −0.23, *P*<.001, *d*=2.05) followed by the number of use events (beta=−.51, CI −0.68 to −0.34, *P*<.001, *d*=1.21; see Multimedia Appendix 3). The proportion of participants with cannabis dependence dropped from 98.5% during baseline to 78.4% (3 months), 67.0% (6 months), and 62.6% (12 months).

**Table 3:** Participant characteristics at baseline and usage of the intervention.

Characteristics		Factor 1: Chat-based communication		Factor 2: Length			All participants (n=534)	
		No (n=263)	Yes (n=271)		28 days (n=266)	50 days (n=268)		
**Intervention versions**		3, 4	1, 2		2, 4	1, 3		1, 2, 3, 4
**Gender**								
	Female, n (%)	85 (32.3)	98 (36.2)		91 (34.2)	92 (34.3)		183 (34.3)
	Male, n (%)	178 (67.7)	173 (63.8)		175 (65.8)	176 (65.7)		351 (65.7)
	Age, mean (SD)	27.5 (7.3)	27.6 (6.7)		28.2 (7.1)	26.8 (6.8)		27.5 (7.0)
**Educational level, n (%)**								
	Basic school (Hauptschule)	25 (9.5)	29 (10.7)		30 (11.3)	24 (9.0)		54 (10.1)
	Middle school (Realschule)	64 (24.3)	57 (21.0)		61 (22.9)	60 (22.4)		121 (22.7)
	High school (Gymnasium)	165 (62.7)	181 (66.8)		167 (62.8)	179 (66.8)		346 (64.8)
	Other school	9 (3.4)	4 (1.5)		8 (3.0)	5 (1.9)		13 (2.4)
**Cannabis**								
	Use days^a^, mean (SD)	24.7 (7.3)	25.1 (6.5)		24.9 (7.0)	24.9 (6.8)		24.9 (6.9)
	Use events^a^, mean (SD)	122.5 (111.6)	120.1 (104.1)		123.7 (108.9)	118.9 (106.8)		121.2 (107.7)
	Quantity (grams)^a^, mean (SD)	23.2 (18.8)	21.3 (18.6)		23.2 (19.6)	21.3 (17.8)		22.2 (18.7)
	SDS^b,c^, mean (SD)	9.9 (2.8)	10.0 (2.7)		10.1 (2.5)	9.8 (2.9)		10.0 (2.7)
	SDS^b,c^ cannabis dependence, n (%)	260 (98.9)	266 (98.2)		265 (99.6)	261 (97.4)		526 (98.5)
	Currently no professional help, n (%)	196 (74.4)	217 (80.1)		206 (77.4)	207 (77.2)		413 (77.3)
**Usage of the intervention, mean (SD)**								
	Number of logins	25.1 (26.4)	29.7 (27.2)		23.2 (20.8)	31.6 (31.2)		27.4 (26.9)
	Chat-based counseling (min)	3.2 (30.1)	105.3 (43.8)		53.8 (59.9)	56.8 (66.9)		55.3 (63.5)

^a^During the past 30 days.

^b^SDS: Severity of Dependence Scale.

^c^Cutoff of ≥4 for cannabis dependence [17].

**Table 4:** Outcome scores at all 4 study points.

Characteristics		Factor 1: Chat-based communication^a^		Factor 2: Length^a^		
		No (n=263)	Yes (n=271)		28 days (n=266)	50 days (n=268)
**Cannabis use^b^ (days), mean (SD)**						
	Baseline	24.7 (7.3)	25.1 (6.5)		24.9 (7.0)	24.9 (6.8)
	3 months	8.1 (9.4)	7.6 (9.6)		8.6 (9.9)	7.1 (9.1)
	6 months	8.5 (9.7)	8.5 (9.9)		9.4 (10.2)	7.6 (9.3)
	12 months	9.6 (10.3)	9.1 (10.3)		10.3 (10.6)	8.4 (9.9)
**Cannabis use^b^ (number of events), mean (SD)**						
	Baseline	122.5 (111.6)	120.1 (104.1)		123.7 (108.9)	118.9 (106.8)
	3 months	25.2 (43.5)	20.3 (35.7)		25.3 (44.3)	20.1 (35.0)
	6 months	29.1 (48.9)	25.5 (45.0)		30.4 (49.8)	24.2 (44.1)
	12 months	33.9 (55.2)	29.3 (51.1)		35.2 (56.4)	27.9 (49.5)
**Cannabis use^b^ (grams), mean (SD)**						
	Baseline	23.2 (18.8)	21.3 (18.6)		23.2 (19.6)	21.3 (17.8)
	3 months	6.4 (9.8)	5.0 (8.2)		6.3 (9.7)	5.2 (8.2)
	6 months	6.5 (9.5)	5.5 (8.7)		6.9 (10.1)	5.1 (8.0)
	12 months	7.3 (10.6)	6.8 (10.4)		7.6 (10.8)	6.5 (10.1)
**Cannabis dependence (SDS^c^), mean (SD)**						
	Baseline	9.9 (2.8)	10.0 (2.7)		10.1 (2.5)	9.8 (2.9)
	3 months	7.2 (3.5)	6.8 (3.6)		7.0 (3.5)	6.9 (3.6)
	6 months	5.4 (3.5)	5.1 (3.8)		5.4 (3.6)	5.1 (3.7)
	12 months	5.5 (3.6)	5.4 (3.8)		5.7 (3.6)	5.2 (3.8)
**Working alliance (WAI-sr^d^), mean (SD)**						
	3 months	3.3 (1.0)	3.5 (0.9)		3.5 (0.9)	3.3 (1.0)
**Treatment satisfaction (CSQ-8^e^), mean (SD)**						
	3 months	1.8 (0.7)	2.1 (0.7)		2.0 (0.6)	1.9 (0.7)

^a^Intention-to-treat analyses following multiple imputation. Results of the nonimputed datasets can be found in Multimedia Appendix 3.

^b^During the past 30 days.

^c^SDS: Severity of Dependence Scale.

^d^WAI-sr: Working Alliance Inventory-short revised.

^e^CSQ: Client Satisfaction Questionnaire.

**Table 5:** Group comparisons and interactions between both factors.

Characteristics		Group difference: chat no versus yes^a,b^			Group difference: 28 versus 50 days^a,b^			Interaction factor 1×factor 2×time^a,c^	
		Beta (95% CI)	*P* value	Effect size *d* (95% CI)	Beta (95% CI)	*P* value	Effect size *d* (95% CI)	Beta (95% CI)	*P* value
**Cannabis use^d^ (days), mean (SD)**									
	Baseline	−.02 (−0.14 to 0.10)	.74	N/A^e^	−.09 (−0.23 to 0.05)	.20	N/A	−.01 (−0.18 to 0.16)	.91
	3 months	N/A	N/A	0.10 (−0.07 to 0.27)	N/A	N/A	0.15 (−0.02 to 0.32)	N/A	N/A
	6 months	N/A	N/A	0.07 (−0.10 to 0.24)	N/A	N/A	0.18 (0.01 to 0.35)	N/A	N/A
	12 months	N/A	N/A	0.10 (−0.07 to 0.27)	N/A	N/A	0.17 (0.00 to 0.35)	N/A	N/A
**Cannabis use^d^ (number of events), mean (SD)**									
	Baseline	−.05 (−0.25 to 0.16)	.64	N/A	−.09 (−0.31 to 0.13)	.43	N/A	−.03 (−0.30 to 0.24)	.82
	3 months	N/A	N/A	0.10 (−0.07 to 0.27)	N/A	N/A	0.09 (−0.08 to 0.26)	N/A	N/A
	6 months	N/A	N/A	0.05 (−0.12 to 0.22)	N/A	N/A	0.09 (−0.08 to 0.26)	N/A	N/A
	12 months	N/A	N/A	0.06 (−0.11 to 0.23)	N/A	N/A	0.09 (−0.08 to 0.26)	N/A	N/A
**Cannabis use^d^ (grams), mean (SD)**									
	Baseline	.01 (−0.17 to 0.19)	.88	N/A	−.04 (−0.23 to 0.15)	.70	N/A	−.05 (−0.27 to 0.18)	.69
	3 months	N/A	N/A	0.06 (−0.11 to 0.23)	N/A	N/A	0.02 (−0.15 to 0.19)	N/A	N/A
	6 months	N/A	N/A	0.00 (−0.17 to 0.17)	N/A	N/A	0.09 (−0.08 to 0.26)	N/A	N/A
	12 months	N/A	N/A	−0.06 (−0.23 to 0.11)	N/A	N/A	0.01 (−0.16 to 0.18)	N/A	N/A
**Cannabis dependence (SDS^f^), mean (SD)**									
	Baseline	−.02 (−0.09 to 0.05)	.63	N/A	−.02 (−0.0 to 0.05)	.57	N/A	.01 (−0.09 to 0.10)	.91
	3 months	N/A	N/A	0.14 (−0.03 to 0.31)	N/A	N/A	−0.10 (−0.27 to 0.07)	N/A	N/A
	6 months	N/A	N/A	0.11 (−0.06 to 0.28)	N/A	N/A	−0.04 (−0.21 to 0.13)	N/A	N/A
	12 months	N/A	N/A	0.06 (−0.11 to 0.23)	N/A	N/A	0.00 (−0.17 to 0.17)	N/A	N/A
**Working alliance (WAI-sr^g^), mean (SD)**									
	3 months	.36 (0.09 to 0.63)	.008	0.22 (0.05 to 0.39)	−.05 (−0.32 to 0.22)	.71	−0.21 (−0.38 to −0.04)	−.27 (−0.64 to 0.09)	.15
**Treatment satisfaction (CSQ-8>^h^), mean (SD)**									
	3 months	.33 (0.14 to 0.52)	.001	0.34 (0.17 to 0.51)	−.03 (−0.21 to 0.16)	.76	−0.17 (−0.34 to 0.00)	−.16 (−0.42 to 0.10)	.22

^a^Intention-to-treat analyses following multiple imputation. Results of the nonimputed datasets can be found in Multimedia Appendix 3.

^b^Between-group comparisons were conducted with the interaction of each factor with time, except for the effects on WAI-sr and CSQ-8 which were analyzed with the main effect of each factor.

^c^Effects on WAI-sr and CSQ-8 were analyzed with the interaction between factor 1 and factor 2.

^d^During the past 30 days.

^e^N/A: not applicable.

^f^SDS: Severity of Dependence Scale.

^g^WAI-sr: Working Alliance Inventory-short revised.

^h^CSQ-8: Client Satisfaction Questionnaire.

## Discussion

### Principal Findings

This study examined whether the effectiveness of the internet intervention QTS is reduced by removing chat-based counseling or by shortening the program. To our knowledge, it is the first trial designed to systematically analyze these key characteristics of guided internet interventions. According to the results, neither of these changes has meaningful impact on the effectiveness of QTS. As study participants were comparable to nonparticipants, we assume results can be generalized to regular users of QTS.

The results correspond to outcomes of meta-analyses, which also found no effects of changes in program duration on the outcomes of internet interventions for substance users [6,33,34]. In QTS, similar results of the shorter and longer program versions might be explained by a relatively fast onset of effects in the first weeks of participation and by a stabilization of these effects afterwards. Similar developments of use-related outcomes were found in another trial about an internet intervention for cannabis users [35].

The similar effects of the longer versions might also be related to a disproportionally low user engagement in these versions, a pervasive phenomenon coined as the law of attrition [36]. Therefore, the increment of received support was probably too small to significantly enhance the effects as compared with the shorter program versions.

The similar performance of the nonchat-based program versions corresponds with results of an earlier meta-analysis, which found no effectiveness difference between synchronous and asynchronous communication [13]. Our results suggest that the removal of chat-based counseling can be compensated by other elements of QTS, like the self-guided tour at the beginning or by enriching the weekly feedbacks with more information. However, extended feedbacks might not be sufficient to compensate a lack of an effective intervention element, as results from another study in this field of research suggest [37]. In contrast to our results, Schaub et al [35] found additional effects of chat counseling in a Web-based intervention for cannabis users. One key reason for the differences between their and our findings may be due to the reference conditions: the nonchat-group (active control) in the Swiss study received an automated self-help program, whereas the nonchat-conditions in our study included therapist guidance. Therefore, in our study, the chat and nonchat groups were probably more similar in terms of received support than the corresponding group in the study of Schaub and colleagues.

In contrast to the nonsignificant cannabis-related outcomes, we found a stronger working alliance and higher satisfaction ratings among users of the chat-based versions. These results are in line with outcomes of an earlier comparison study [38], supporting the assumption that direct interaction leads to a closer cooperation between the client and counselor and thus to better satisfaction ratings. These findings, therefore, should be taken into account in future modifications of QTS.

With a within-group effect size of *d*=2.05 between study baseline and the 3-month follow-up, study participants strongly reduced the frequency of their cannabis use. This effect surpasses the reductions found among QTS participants in our earlier trial (within-group *d*=1.47 for use frequency) [4,39] and also goes beyond the effects found among users of the Web-based intervention with chat counseling studied by Schaub et al (within-group *d*=0.75 for use frequency) [35] and the reductions found in the self-guided treatment examined by Rooke and colleagues (within-group *d*=1.08 for use frequency) [40]. Although within-group changes should always be interpreted carefully, the strong overall reductions in this study presumably reflect the high level of support provided by QTS.

### Strengths and Limitations

We took several measures to ensure validity of the study results. We strictly adhered to the CONSORT rules, implemented a randomized factorial study design, tested and controlled potential confounders in the analyses, and compared results from the main analyses with those of completer-only analyses. Furthermore, the original intervention was already successfully tested in a randomized study [4] and is conducted by qualified staff with several years’ experiences in online counseling.

As in other studies in this field of research [4,35,40,41], a major weakness is the low follow-up rate. Although we applied multiple imputations on the dataset, validity of the longer-term results is probably reduced. However, results are coherent across follow ups and, except for the significant interaction on the WAI and CSQ score, across datasets. Nevertheless, future studies in this field of research should look for ways of decreasing participant attrition. Compared with our earlier trial on the effectiveness of QTS [4], we were able to increase follow-up rates significantly. We suppose this mainly goes back to improvements in the follow up-recruitment, like offering shopping vouchers for each follow-up, emphasizing the short duration of each questionnaire, repeatedly inviting every participant for each follow-up, and addressing each nonresponder personally.

As all purely Web-based RCTs, we relied on self-reported data. This poses a threat to validity, especially for the cannabis-related outcomes, as cannabis use still is illegal in Germany and therefore might be understated by the participants. However, we deemed a biological validation of these outcomes unfeasible, as the collection of biological markers is associated with high costs, a low measurement precision, and a narrow selection of participants to those who are willing to provide these data. It must also be noted that the outcomes “quantity of cannabis” and “number of use events” did not account for the type of cannabis product (eg, hashish, marijuana), its administration (eg, joint, bong), or its THC content. Therefore, the measurement precision of these outcomes is probably reduced. Other studies aim to improve the estimation by a standardization formula [37,40] or by gaining more detailed information on the cannabis product typically used [35]. Despite their apparent advantages, we chose not to use either of these procedures as they are not validated for application in the German setting.

### Conclusions

The reduction of program length and the waiver of synchronous communication have no meaningful impact on the effectiveness of QTS. It therefore seems tenable to abbreviate the program and to offer a self-guided start into QTS. As chat-based counseling shows higher user ratings, it should be provided for those users who prefer to be supported that way.

## Appendix

Multimedia Appendix 1 Information for trial participants.

Multimedia Appendix 2 Screenshot of the program diary.

Multimedia Appendix 3 Additional Tables.

Multimedia Appendix 4 CONSORT‐EHEALTH checklist (V 1.6.1).

## Abbreviations

BZgA: Bundeszentrale für gesundheitliche Aufklärung, Federal Centre for Health Education
CUD: cannabis use disorder
CSQ-8: Client Satisfaction Questionnaire
RCT: randomized controlled trial
QTS: Quit the Shit
SDS: Severity of Dependence Scale
TLFB: Timeline Followback
WAI-sr: Working Alliance Inventory-short revised
