# Supplementary material for: Effects of Treatment Length and Chat-Based Counseling in a Web-Based Intervention for Cannabis Users: Randomized Factorial Trial
Source: J Med Internet Res. 2018 May 8;20(5):e166. doi: 10.2196/jmir.9579 (PMC5964299; doi:10.2196/jmir.9579)
Supplement: Multimedia Appendix 1 [file jmir_v20i5e166_app1.pdf]

Informationen zur Studie

Bevor du dich für oder gegen eine Teilnahme an der aktuellen Studie entscheidest, möchten wir dir das Ziel und den Ablauf der Studie erläutern. Wenn du hierzu Fragen hast, stehen wir dir gerne zur Verfügung. Unsere Kontaktdaten findest du unten.

Was ist das Ziel der Studie?

In der Studie wollen wir herausfinden, wie das Programm seinen Nutzerinnen und Nutzern hilft, ihren Cannabiskonsum zu reduzieren. Wir wollen also herausfinden, wie gut das Programm funktioniert. Zudem möchten wir wissen, welche Wirkung einzelne Teile des Programms haben.

In der Studie vergleichen wir deswegen verschiedene Versionen des Programms, deren Länge und Inhalt jeweils unterschiedlich sind. Alle Teilnehmerinnen und Teilnehmer bekommen unabhängig von der Version des Programms

- mindestens vier Wochen Zugang zum persönlichen Konsumtagebuch und wöchentlich individuelle Rückmeldungen vom Beratungsteam,
- die Möglichkeit, die Übungen des Programms zu nutzen und
- die Möglichkeit, am Forum von Quit the Shit teilzunehmen.

Was passiert in der Studie?

Wenn du an der Studie teilnimmst, wirst du einer Variante des Programms automatisch zugelost. Egal, welcher Version du zugeordnet wirst: Du kannst Quit the Shit nutzen und erhältst somit anonyme, kostenlose und kompetente Beratung.

Zur Studie gehören insgesamt drei Nachbefragungen. Hierzu wirst du 3 Monate, 6 Monate und 12 Monate nach Programmeinstieg per E-Mail eingeladen. Die Nachbefragungen werden per Online-Fragebogen durchgeführt.

Die Nachbefragungen sind für die Studie sehr wichtig. Daher erhältst du pro Nachbefragung (ca. 10 min) einen Einkaufsgutschein von amazon.de im Wert von 10 Euro, für alle drei Nachbefragungen also **Warengutscheine über insgesamt 30 Euro**.

Wie geht es weiter?

- **Am Anfang: Die Anmeldung.** Hierzu gehört ein Anmelde-Fragebogen, der komplett ausgefüllt werden muss. Hiermit wird unter anderem geprüft, ob du die nötigen Voraussetzungen zur Teilnahme an der Studie erfüllst. Erfüllst du sie nicht, kannst du trotzdem ins Programm einsteigen, wirst aber nicht nachbefragt. Am Ende der Anmeldung musst du dir noch einen Termin für den Einstieg ins Programm reservieren. Die Anmeldung plus Terminreservierung dauert rund **20-30 Minuten**.
- **Ein paar Tage später** erfolgt dein Einstieg in Quit the Shit. Hierzu loggst du dich an deinem gewählten Termin ein und beginnst mit deiner Programmteilnahme. Der Termin dauert **60 Minuten**.
- **Das Programm:** Das Kernstück des Programms ist das **Tagebuch**, wo du alle wesentlichen Dinge, die mit deinem Cannabiskonsum zusammenhängen, festhältst. Du kannst das Tagebuch mindestens vier Wochen lang nutzen. Darüber hinaus enthält das Programm verschiedene **Übungs-Module**, die dich dabei unterstützen, deinen Konsum in kritischen Situationen zu kontrollieren. Einmal pro Woche erhältst du eine **detaillierte Rückmeldung von deiner Beraterin oder deinem Berater** auf deine Einträge im Tagebuch und zu den Übungen.

- **In 3, 6 und 12 Monaten** erfolgen die Nachbefragungen per Online-Fragebogen. Die Befragungen dauern jeweils rund 10 Minuten. Für jede Nachbefragungs-Teilnahme erhältst du einen Einkaufsgutschein von amazon.de im Wert von 10 Euro. Deine Teilnahme an den Nachbefragungen ist für die Studie sehr wichtig. Daher werden wir dich telefonisch oder per SMS erinnern, wenn du nicht auf die E-Mail-Einladungen reagierst. Deine Telefonnummer wird nur zu diesem Zweck verwendet und stets vertraulich behandelt.

Du kannst deine Studienteilnahme jederzeit und ohne Angaben von Gründen beenden. Schick uns hierfür eine E-Mail mit deinem Nutzernamen an folgende Adresse: forschung@quit-the-shit.net. Deine Kontaktdaten werden dann sofort gelöscht.

Wer kann an der Studie teilnehmen?

Die Studie steht allen Teilnehmerinnen und Teilnehmern von Quit the Shit offen, die folgende Kriterien erfüllen:

- Mindestalter: 18 Jahre
- Keine bisherige Teilnahme an Quit the Shit
- Bereitschaft, in den nächsten 12 Monaten an drei Nachbefragungen mittels Online-Fragebögen teilzunehmen (Dauer jeweils ca. 10 min)
- Aktuell maximal wöchentlicher Konsum anderer illegaler Substanzen
- Aktuell kein Vorliegen psychiatrischer Krankheiten (z.B. psychotische Störung) oder von Alkoholabhängigkeit

Informationen zum Datenschutz

Die Daten werden nur im Rahmen der Datenauswertung und nicht anderweitig verwendet, gespeichert oder weitergegeben. Der Datenschutz hat für uns einen hohen Stellenwert. Daher gelten für den Umgang mit den Klientendaten strenge Regeln. Diese lauten im Einzelnen:

- **Vertraulicher Umgang mit den Daten**
  Für die Studienanmeldung ist die Angabe deiner Handynummer und E-Mail-Adresse notwendig. Diese Daten werden getrennt von deinen restlichen Angaben in einer verschlüsselten Datei (AES-Verschlüsselung, 256-Bit Schlüssel) auf dem Server von www.quit-the-shit.net gespeichert. Sie werden ausschließlich dazu verwendet, um dich telefonisch oder per SMS an eine fällige Nachbefragung zu erinnern. Sie werden nicht an Dritte weiter gegeben und nach Abschluss deiner Studienteilnahme (d.h. spätestens 12 Monate nach deinem Programmeinstieg) wieder gelöscht. Alle beteiligten Fachkräfte verpflichten sich zur Verschwiegenheit in Bezug auf deine Programmteilnahme.
- **Anonymisierte Datenauswertung**
  Für die Studie werden nur deine Angaben in den Online-Fragebögen und anonymisierte Nutzungszahlen, wie etwa die Nutzungsdauer oder die Zahl der Logins, ausgewertet. Diese Daten werden nicht zusammen mit deinen Kontaktdaten ausgewertet, sodass kein Rückschluss auf deine Person möglich ist. Einträge im Tagebuch und in den Übungen sowie sämtliche Interaktion mit dem Beratungspersonal werden nicht ausgewertet. Die IP-Adresse deines Internet-Anschlusses wird nur anonymisiert in verkürzter Form gespeichert, sodass kein Rückschluss auf deine Person möglich ist.
- **Sichere Datenübermittlung**
  Die Übermittlung deiner Daten im Internet erfolgt ausschließlich verschlüsselt. Dabei kommt eine Technik zum Einsatz, die zum Beispiel auch beim Online-Banking eingesetzt wird (SSL-Verschlüsselung).

- Deine Angaben werden nur im Rahmen der Datenauswertung und nicht anderweitig verwendet, gespeichert oder weitergegeben. Darüber hinaus gelten die Datenschutzbestimmungen von drugcom.de. Du kannst sie unter folgender Adresse einsehen: http://www.drugcom.de/datenschutz/

Noch Fragen?

Bei Fragen zur Studie kannst du uns telefonisch und per E-Mail erreichen.
Studienbetreuung: Dipl.-Psych. Benjamin Jonas (Delphi-Gesellschaft, 14057 Berlin)
Telefon: 030-39 40 97 82 (Bürozeiten Mo.-Fr. 10 bis 18 Uhr)
E-Mail: forschung@quit-the-shit.net
