# Supplementary figures and images for: Effects of Treatment Length and Chat-Based Counseling in a Web-Based Intervention for Cannabis Users: Randomized Factorial Trial
Source: J Med Internet Res. 2018 May 8;20(5):e166. doi: 10.2196/jmir.9579 (PMC5964299; doi:10.2196/jmir.9579)

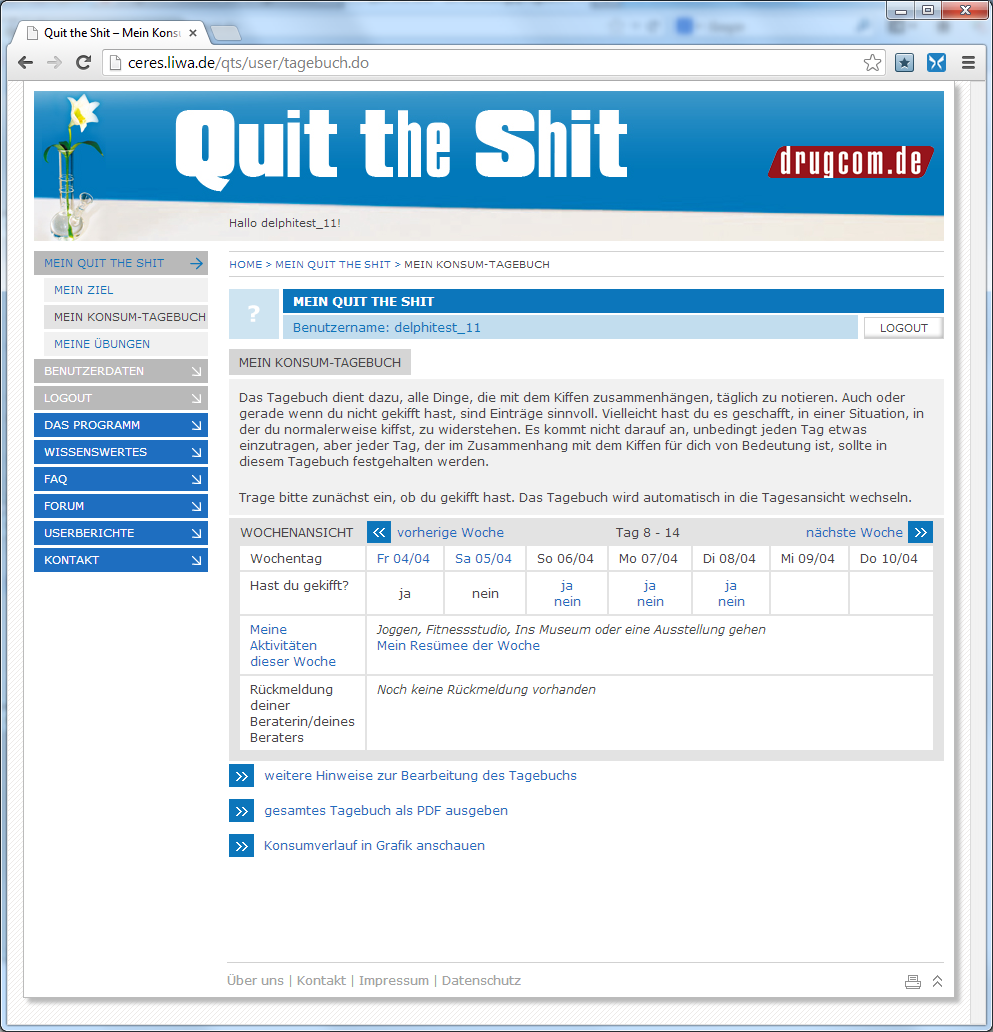

Supplement: Multimedia Appendix 2 [file jmir_v20i5e166_app2.png]
