# Supplementary material for: Effects of Treatment Length and Chat-Based Counseling in a Web-Based Intervention for Cannabis Users: Randomized Factorial Trial
Source: J Med Internet Res. 2018 May 8;20(5):e166. doi: 10.2196/jmir.9579 (PMC5964299; doi:10.2196/jmir.9579)
Supplement: Multimedia Appendix 3 [file jmir_v20i5e166_app3.pdf]

# Multimedia Appendix – Additional Tables

## Table 5. Outcome scores at all four study points.[a]

| | Factor 1: Chat-based communication | | Factor 2: Length | |
|---|---|---|---|---|
| | **no** | **yes** | **28 days** | **50 days** |
| **Cannabis use (days)[b,c]** | | | | |
| **Baseline (n=534)** | 24.7 (7.3) | 25.1 (6.5) | 24.9 (7.0) | 24.9 (6.8) |
| **3 months (n=252)** | 8.4 (9.7) | 7.8 (10.2) | 8.9 (10.4) | 7.4 (9.5) |
| **6 months (n=204)** | 8.8 (10.1) | 8.5 (10.5) | 10.2 (11.0) | 7.4 (9.5) |
| **12 months (n=135)** | 10.3 (11.3) | 8.3 (11.3) | 10.8 (12.0) | 8.0 (10.6) |
| **Cannabis use (number of events)[b,c]** | | | | |
| **Baseline (n=534)** | 122.2 (111.6) | 120.1 (104.1) | 123.4 (108.9) | 118.9 (106.8) |
| **3 months (n=252)** | 22.3 (33.2) | 17.2 (27.8) | 21.3 (32.0) | 18.3 (29.5) |
| **6 months (n=204)** | 27.6 (45.7) | 24.3 (43.0) | 30.4 (46.6) | 22.5 (42.3) |
| **12 months (n=135)** | 36.3 (56.2) | 26.8 (49.2) | 38.7 (58.2) | 25.3 (47.2) |
| **Cannabis quantity (grams)[b,c]** | | | | |
| **Baseline (n=534)** | 23.1 (18.7) | 21.3 (18.6) | 23.1 (19.6) | 21.3 (17.8) |
| **3 months (n=252)** | 5.8 (8.7) | 3.7 (5.4) | 5.2 (7.8) | 4.3 (6.7) |
| **6 months (n=204)** | 5.6 (7.9) | 4.9 (7.5) | 6.4 (9.1) | 4.4 (6.2) |
| **12 months (n=135)** | 6.1 (9.2) | 6.6 (10.4) | 7.4 (10.7) | 5.6 (9.1) |
| **Cannabis dependence (SDS)[b]** | | | | |
| **Baseline (n=534)** | 9.9 (2.8) | 10.0 (2.7) | 10.1 (2.5) | 9.8 (2.9) |
| **3 months (n=252)** | 7.0 (3.6) | 6.5 (3.6) | 6.7 (3.3) | 6.8 (3.8) |
| **6 months (n=204)** | 5.3 (3.4) | 5.0 (4.0) | 5.4 (3.5) | 4.9 (3.8) |
| **12 months (n=135)** | 5.3 (3.7) | 5.2 (4.3) | 5.9 (3.7) | 4.7 (4.2) |
| **Working alliance (WAI-sr)[b]** | | | | |
| **3 months (n=252)** | 3.6 (1.0) | 3.8 (0.8) | 3.7 (0.9) | 3.6 (0.9) |
| **Treatment satisfaction (CSQ-8)[b]** | | | | |
| **3 months (n=252)** | 2.1 (0.7) | 2.3 (0.6) | 2.2 (0.6) | 2.2 (0.6) |

[a] Results of the non-imputed dataset.
[b] Mean (SD)
[c] During the past 30 days.

## Table 6. Group comparisons and interactions between both factors.[a]

| | Group difference: Chat no vs. yes[b] | | | Group difference: 28 vs. 50 days[b] | | | Interaction Factor 1 x Factor 2 x Time[e] | |
|---|---|---|---|---|---|---|---|---|
| | **Beta (95% CI)** | **P value** | **Effect size d (95% CI)** | **Beta (95% CI)** | **P value** | **Effect size d (95% CI)** | **Beta (95% CI)** | **P value** |
| **Cannabis use (days)[c,d]** | | | | | | | | |
| **Baseline** | -0.02 (-0.21; 0.17) | .83 | | -0.10 (-0.29; 0.09) | .29 | | -0.11 (-0.39; 0.17) | .45 |
| **3 months** | | | 0.12 (-0.14 to 0.37) | | | 0.15 (-0.11 to 0.40) | | |
| **6 months** | | | 0.09 (-0.19 to 0.37) | | | 0.26 (-0.02 to 0.54) | | |
| **12 months** | | | 0.23 (-0.12 to 0.59) | | | 0.25 (-0.11 to 0.60) | | |
| **Cannabis use (number of events)[c,d]** | | | | | | | | |
| **Baseline** | -0.03 (-0.37; 0.31) | .86 | | -0.10 (-0.40; 0.21) | .53 | | -0.19 (-0.64; 0.26) | .41 |
| **3 months** | | | 0.15 (-0.11 to 0.40) | | | 0.06 (-0.20 to 0.31) | | |
| **6 months** | | | 0.05 (-0.23 to 0.33) | | | 0.13 (-0.15 to 0.42) | | |
| **12 months** | | | 0.16 (-0.19 to 0.51) | | | 0.21 (-0.14 to 0.56) | | |
| **Cannabis use (grams)[c,d]** | | | | | | | | |
| **Baseline** | 0.05 (-0.23; 0.33) | .73 | | -0.06 (-0.33; 0.21) | .65 | | -0.11 (-0.49; 0.28) | .59 |
| **3 months** | | | 0.19 (-0.06 to 0.45) | | | 0.02 (-0.23 to 0.28) | | |
| **6 months** | | | 0.00 (-0.28 to 0.27) | | | 0.16 (-0.12 to 0.44) | | |
| **12 months** | | | -0.14 (-0.49 to 0.20) | | | 0.08 (-0.27 to 0.43) | | |
| **Cannabis dependence (SDS)[c]** | | | | | | | | |

| | | | | | | | | |
|---|---|---|---|---|---|---|---|---|
| **Baseline** | -0.03 (-0.12; 0.07) | .56 | | -0.06 (-0.15; 0.04) | .23 | | 0.03 (-0.11; 0.17) | .66 |
| **3 months** | | | 0.17 (-0.08 to 0.43) | | | -0.15 (-0.41 to 0.10) | | |
| **6 months** | | | 0.12 (-0.16 to 0.40) | | | 0.00 (-0.28 to 0.28) | | |
| **12 months** | | | 0.04 (-0.31 to 0.39) | | | 0.17 (-0.18 to 0.52) | | |
| **Working alliance (WAI-sr)[c]** | | | | | | | | |
| **3 months** | 0.49 (0.16; 0.83) | .004 | 0.23 (-0.03 to 0.49) | 0.21 (-0.12; 0.53) | .22 | -0.12 (-0.37 to 0.14) | -0.55 (-1.00; -0.10) | .02 |
| **Treatment satisfaction (CSQ-8)[c]** | | | | | | | | |
| **3 months** | 0.47 (0.24; 0.69) | <.001 | 0.41 (0.16 to 0.67) | 0.19 (-0.03; 0.42) | .09 | -0.05 (-0.31 to 0.20) | -0.41 (-0.72; -0.10) | .01 |

[a] ITT analyses of the non-imputed dataset.

[b] Between-group comparisons were conducted with the interaction of each factor with time, except for the effects on WAI-sr and CSQ-8 which were analyzed with the main effect of each factor.

[c] Mean (SD)

[d] During the past 30 days.

[e] Effects on WAI-sr and CSQ-8 were analyzed with the interaction between Factor 1 and Factor 2

**Table 7. Per-group-scores of working alliance & treatment satisfaction in the non-imputed dataset**

**Working alliance (WAI-sr), Mean (SD)**

| | | **Factor 2: Intervention length** | |
|---|---|---|---|
| | | **50 days** | **28 days** |
| **Factor 1: Chat-based counselling** | **yes** | 3.6 (0.9) (n=68) | 4.0 (0.7) (n=58) |
| | **no** | 3.6 (1.0) (n=67) | 3.5 (1.0) (n=59) |

**Treatment satisfaction (CSQ-8), Mean (SD)**

| | | **Factor 2: Intervention length** | |
|---|---|---|---|
| | | **50 days** | **28 days** |
| **Factor 1: Chat-based counselling** | **yes** | 2.2 (0.7) (n=68) | 2.4 (0.5) (n=58) |
| | **no** | 2.1 (0.7) (n=67) | 2.0 (0.7) (n=59) |

**Table 8. Within-group effect sizes[a]**

| | Main effects of time[b] | | | Main effects of time[b] | | |
|---|---|---|---|---|---|---|
| | **Beta (95% CI)** | **P value** | **Effect size d** | **Beta (95% CI)** | **P value** | **Effect size d** |
| **Cannabis use (days)[d,e]** | -0.34 (-0.45; -0.23) | < .001 | 1.92 | -0.40 (-0.52; -0.27) | < .001 | 2.03 |
| **Cannabis use (number of events)[d,e]** | -0.51 (-0.68; -0.34) | < .001 | 1.14 | -0.65 (-0.87; -0.43) | < .001 | 1.01 |
| **Cannabis use (grams)[d,e]** | -0.46 (-0.61; -0.32) | < .001 | 1.06 | -0.64 (-0.83; -0.44) | < .001 | 1.00 |
| **Cannabis dependence (SDS)[d]** | -0.21 (-0.26; -0.15) | < .001 | 1.16 | -0.23 (-0.29; -0.17) | < .001 | 1.31 |

[a] Difference between Baseline and the 3 months-follow up.
[b] Results of the imputed datasets.
[c] Results of the non-imputed dataset.
[d] Mean (SD)
[e] During the past 30 days.
